# Giant circular dichroism of large-area extrinsic chiral metal nanocrecents

**DOI:** 10.1038/s41598-018-21627-z

**Published:** 2018-02-20

**Authors:** Yane Wang, Jiwei Qi, Chongpei Pan, Qiang Wu, Jianghong Yao, Zongqiang Chen, Jing Chen, Yudong Li, Xuanyi Yu, Qian Sun, Jingjun Xu

**Affiliations:** 1 MOE Key Laboratory of Weak Light Nonlinear Photonics, Tianjin Key Laboratory of Photonics and Technology of Information Science, School of Physics, Nankai University, Tianjin, 300071 China; 2 Collaborative Innovation Center of Extreme Optics, Shanxi University, Taiyuan, Shanxi 030006 China

## Abstract

In this work, we demonstrate the strong extrinsic chirality of the larger-area metal nanocrescents by experiments and simulations. Our results show that the metal nanocrescent exhibits giant and tunable circular dichroism (CD) effect, which is intensively dependent on the incident angle of light. We attribute the giant extrinsic chirality of the metal nanocrescent to the excitation efficiencies difference of localized surface plasmon resonance (LSPR) modes for two kinds of circularly polarized light at a non-zero incident angle. In experiment, the largest CD of 0.37 is obtained at the wavelength of 826 nm with the incident angle of 60°. Furthermore, the CD spectra can be tuned flexibly by changing the metal nanocrescent diameter. Benefitting from the simple, low-cost and mature fabrication process, the proposed large-area metal nanocrescents are propitious to application.

## Introduction

Chirality is a general property, which has taken an extremely important role in biology, chemistry, physics and medicine^1,2^. CD is a manifestation form of chirality, which is due to the difference in extinction for left circularly polarized (LCP) and right circularly polarized (RCP) light. The CD effect opens up novel opportunities in chiral catalysis^3^, chiral discrimination^4^, polarization-sensitive optical devices^5^, sensing^6^, broadband circular polarizers^7^, and three-dimensional display. More and more researchers have paid attention to the chiral materials, such as chiral molecules^8^, hybrid complex of achiral plasmonic nanoparticale and chiral medium^9,10^, metallic nanoparticales^11,12^, chiral metamaterials^13,14^ and so on. Among these applications, chiral metal structure plays a key role due to its giant optical activity and sensitive tunability. The giant optical activity of chiral metal structure origins from the strong interaction between light and surface plasmon resonance (SPR) mode. And the sensitive tunability of the structure results from the huge sensitivity of SPR to the structure size and the surrounding refractive index^15,16^.

Apart from chiral objects, achiral structures can also show the same effect under certain conditions, which is first reported in reference^17^ and later called extrinsic chiral structures^18^. For those achiral structures, the strength of the chiral signal is extremely sensitive to the tilt of the material plane relative to the incident beam^19,20^. Extrinsic chirality has become a hotspot in recent years and lots of extrinsic chiral plasmonic structures have been put forward. In 2008, N. I. Zheludev and his collaborator observed CD effect in GHz region from an array of metal split rings induced by extrinsic chirality^21^. Later, extrinsic chirality structures, such as curved gold metal nanowires and nanorice heterodimers, expanded the induced CD to visible and near-infrared (NIR) region^22,23^. Recently, Leon *et al*. demonstrated experimentally and theoretically strong CD of the extrinsic chiral metasurface consisting of an array of gold split ring resonators^24^. These plasmonic structures with strong extrinsic chiral effects are expected to develop some opportunities in application. As we know, most extrinsic chiral structures were fabricated using electron beam lithography or focused ion beam, which is very expensive and time-consuming to producing the large-area extrinsic chiral structures^25,26^. Fabrication of low-cost large-area chiral and extrinsic chiral plasmonic structures has become a key factor for the application of chiral and extrinsic chiral effect. Nowadays, accompanied by the development of the micro/nanofabrication technologies, multifarious large-area plasmonic structures with low-cost and high-efficiency fabrication have been achieved, such as nanoshell arrays^27^, nanohole array^28^, nanodisk array^28^, nanocrescent^29^ and nanowires^30^. However, research concentrating on the chirality and extrinsic chirality of these low-cost large-area plasmonic structures is still lacking.

Here, we report the observation of giant and tunable extrinsic chirality of the large-area metal nanocrescent structures by experiments and simulations. The large-area and monodisperse nanocrescents are fabricated by low-cost nanosphere lithography (NL) technique^31^. And the metal nanocrescent shows giant and tunable CD responses. The CD peaks appear near the frequency of LSPR bands. Based on these results, we give a qualitative explanation for the giant extrinsic chirality of the metal nanocrescent, that at a non-zero incident angle *θ*, the excitation efficiencies of LSPR modes for the two kinds of circularly polarized light are different. The experimental results are basically consistent with ones in simulations. The maximum CD of 0.37 at around 826 nm is obtained experimentally. At last, we demonstrate that the CD spectra can be tuned flexibly by changing the metal nanocrescent diameter. Our low-cost large-area metal nanocrescents with giant and tunable CD stand a good chance to promote the practical application of chiral effect.

## Results and Discussion

**Figure 1:**
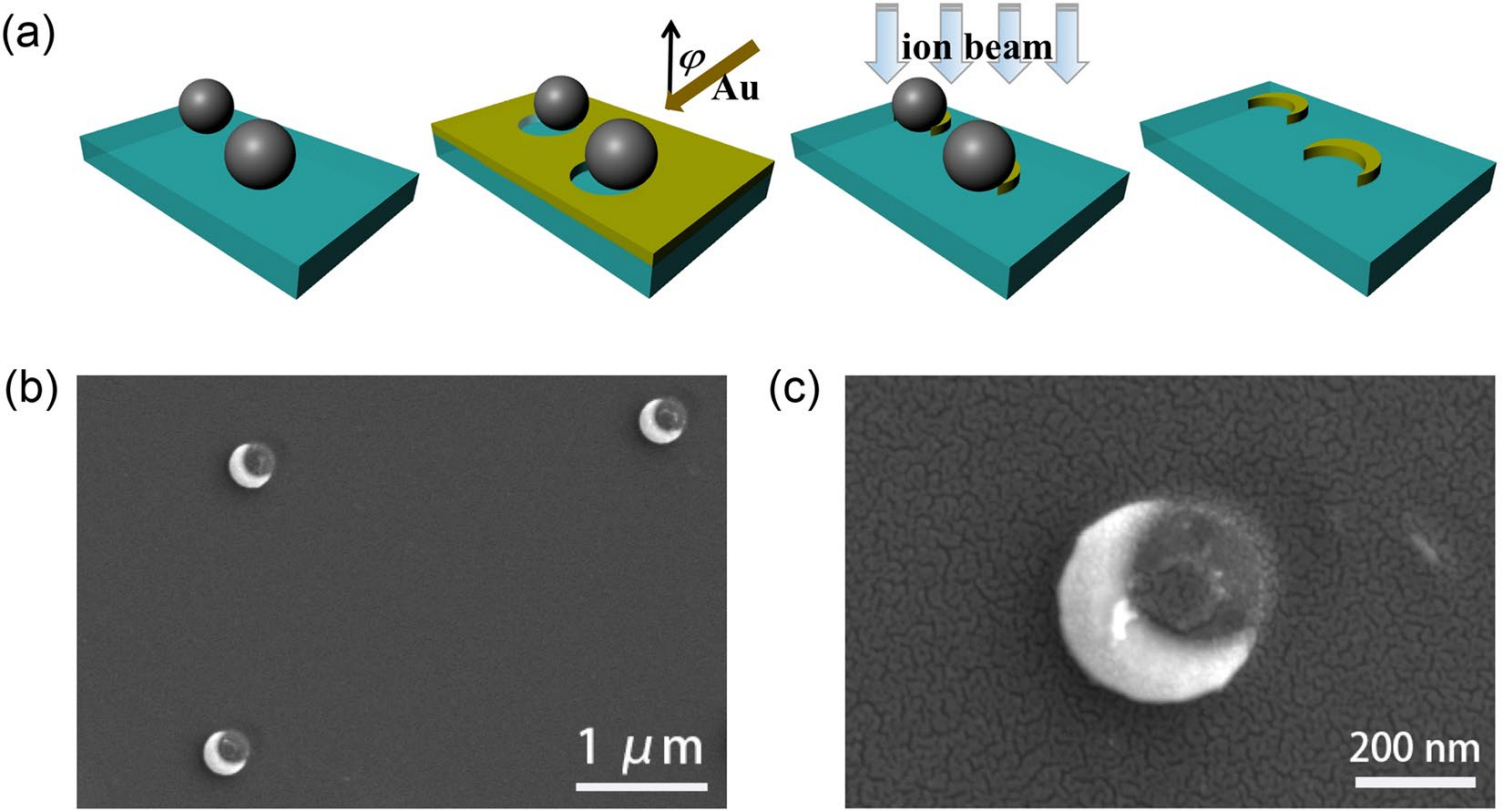
(**a**) Scheme of the metal nanocrescents preparation process. (**b**) SEM image of the metal nanocrescents: dispersed structures show uniformity of shape, size, and orientation from NL fabrication. (**c**) The SEM image of a single metal nanocrescent.

The large-area metal nanocrescents were fabricated using NL technology (see METHODS). Figure 1a shows the scheme of the fabrication process. With this method, we achieve the metal nanocrescents with uniform size, shape, and orientation, whose SEM figure is shown in Fig. 1b. Enlarged detail of the metal nanocrescent is also given in Fig. 1c. The metal nanocrescents are monodisperse distribution on 2 cm × 1 cm substrate with the diameter of 300 nm and the thickness of 50 nm. Here, a tilt angle of $\phi$ is equal to 45°.

**Figure 2:**
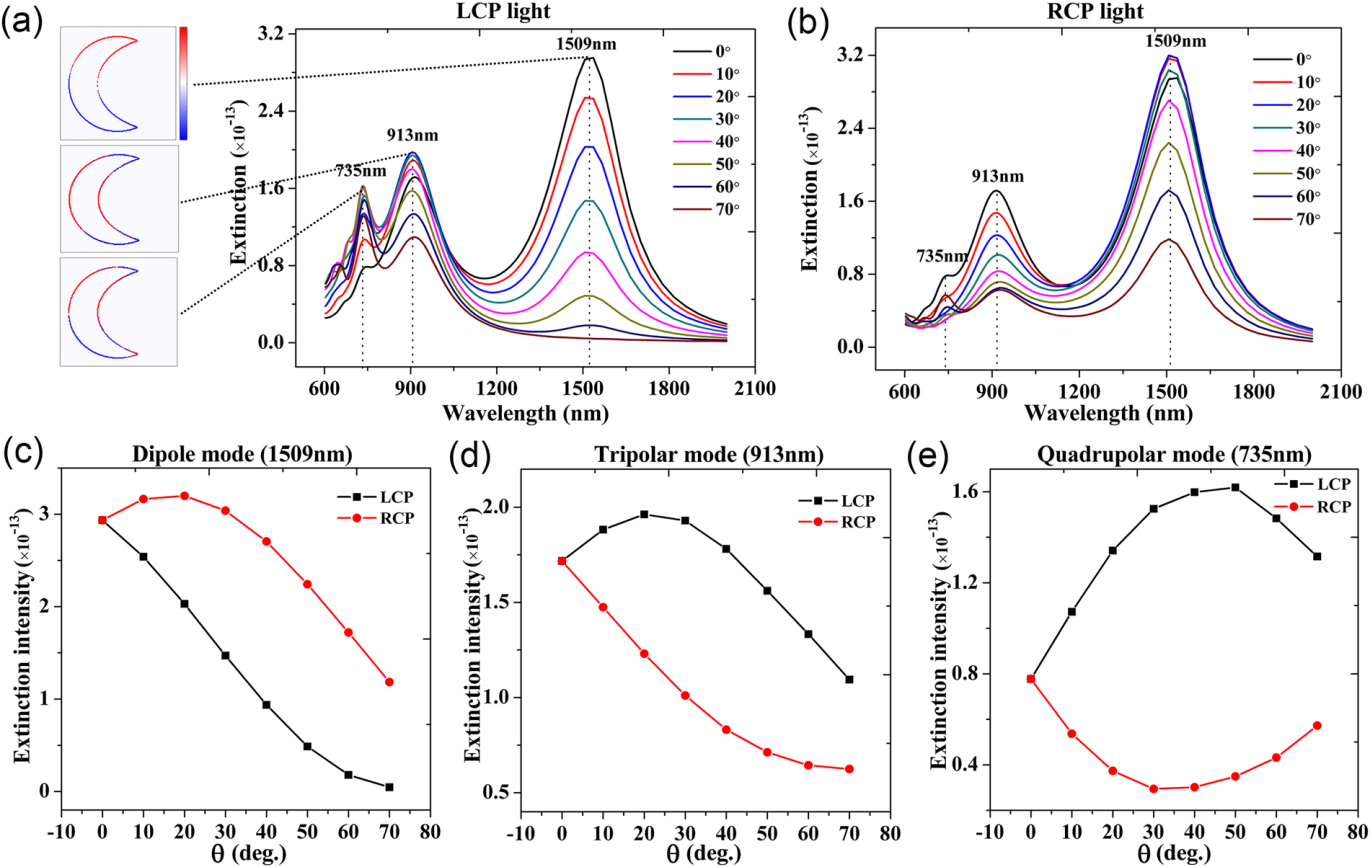
Simulation results of the metal nanocrescent. (**a**) The extinction spectra of the metal nanocrescent for LCP light with the increase of *θ*. The insets show that charge distribution of the metal nanocrescent for different resonance modes when $\theta =0^\circ$. (**b**) The extinction spectra of the metal nanocrescent for RCP light with the increase of *θ*. (**c**–**e**) The dependence of extinction intensities of the metal nanocrescent as the function of *θ* for the LCP light (black line) and RCP light (red line).

We firstly study the extrinsic chirality of nanocrescent using finite-difference time-domain method (Lumerical FDTD Solutions). The metal nanocrescent is modeled using the dimension measured from fabricated nanocrescents. Due to the large distance between the randomly arranged metal nanocrescents (Fig. 1b), we ignore the interaction between adjacent metal nanocrescents and only focus on a single metal nanocrescent. The simulation results are shown in Fig. 2. There are three peaks in the extinction spectra, which are located at around 1509 nm, 913 nm and 735 nm, respectively. The insets in Fig. 2a show the charge distributions of the metal nanocrescent at three resonance peaks mentioned above. The results reveal that the resonances at 1509 nm, 913 nm and 735 nm correspond to dipolar mode, tripolar mode and quadrupolar mode respectively. Our results are consistent with reference^32^.

Figure 2a,b show that the extinction spectra of the metal nanocrescent with different incident angles *θ* for LCP and RCP light respectively. When $\theta =0^\circ$, there is no difference in extinction spectra between LCP and RCP light, which indicates that the nanocrescent is achiral. However, when $\,\theta \ne 0^\circ$, the spectra exhibit distinctly different for LCP and RCP light. For a clearer demonstration, the extinction peak intensities at around 1509 nm (dipole mode), 913 nm (tripolar mode) and 735 nm (quadrupolar mode) as a function of *θ* are plotted for LCP light (blank line) and RCP (red line) light in Fig. 2c–e, respectively. The dipole peak intensity (1509 nm) excited by LCP light shows continuous decrease with the increase of *θ*. On the contrast, the dipole peak intensity excited by RCP light firstly increases and then decreases with the increase of *θ* in Fig. 2c. As shown in Fig. 2d, the tripolar peak intensity (913 nm) excited by the LCP light firstly increases and then decreases with the increase of *θ*. The tripolar peak intensity excited by RCP light is a continuous decrease with the increase of *θ*. Meanwhile, the quadrupolar peak intensity (734 nm) excited by the LCP light increases firstly and then decreases with the increase of *θ*. The quadrupolar peak intensity excited by the RCP light decreases firstly and then increases with the increase of *θ* as shown in Fig. 2e. The vast differences existing in the extinction spectra between LCP and RCP light demonstrate the giant extrinsic chirality of the metal nanocrescent.

**Figure 3:**
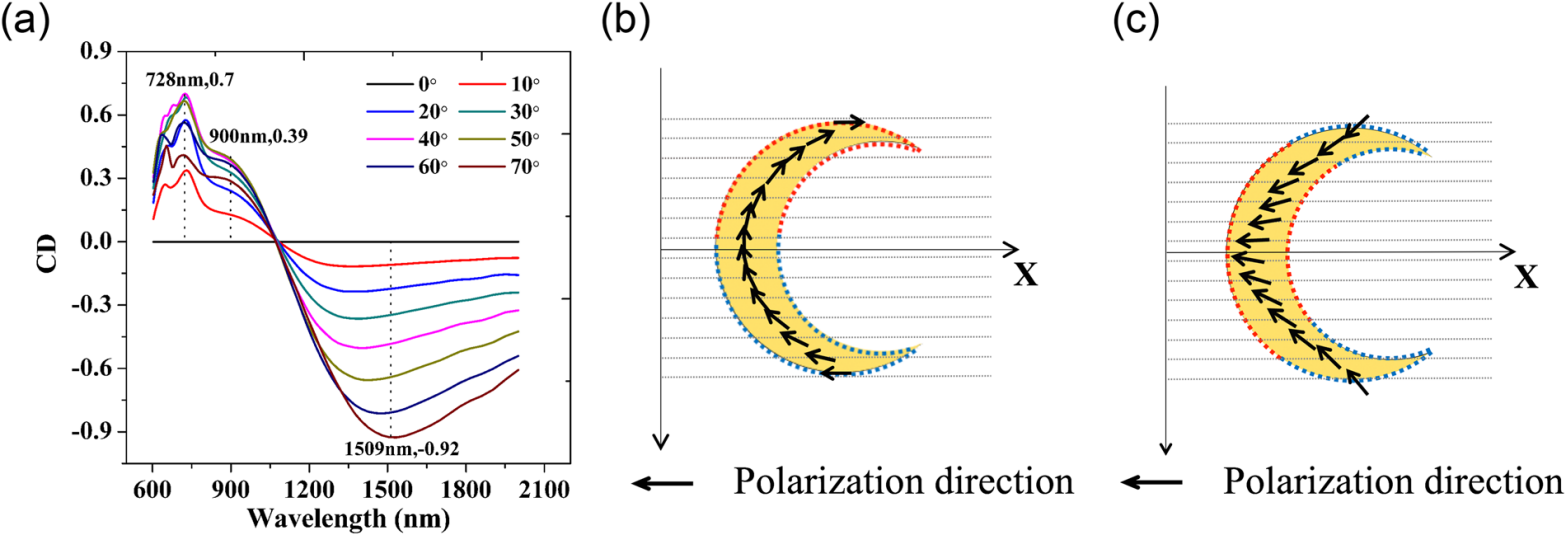
(**a**) The calculated CD spectra of the metal nanocrescent with the increase of *θ*. (**b,c**) Sketch of the optimum electric field distribution on the surface of the nanocrescent of circularly polarized light to excite dipole and tripolar modes, respectively. The black arrows show the polarization direction of pump light. The red dashed lines stand for positive charges and blue dashed lines stand for negative charges.

The CD coefficient can be calculated using the equation below.

,1 $${\rm{CD}}=\frac{{L}_{ext}-{R}_{ext}}{{L}_{ext}+{R}_{ext}}$$

where ${L}_{ext}$ and ${R}_{ext}$ are the extinction intensities of the metal nanocrescent for LCP light and RCP light, respectively. The calculated CD spectra of the metal nanocrescent are shown in Fig. 3a. When $\theta =0^\circ$, CD is equivalent to zero, which suggests that the system is achiral. As *θ* increases, non-zero CD appears. There are three CD peaks in the CD spectra near the frequency of LSPR bands, which are located at around 1509 nm, 900 nm and 728 nm, respectively. The maximum calculated CD are 0.70 at 728 nm, 0.39 at 900 nm and −0.92 at 1509 nm, the absolute values of which are giant.

Based on the simulation results, we give a qualitative explanation of our results and the extrinsic chirality of the metal nanocrescent. In general, the projection area of the metal nanocrescent and the component of the electric field of light on the metal nanocrescent surface are both decreasing with the increase of *θ*, which results in an equivalent decrease of the extinction cross sections for LCP and RCP light. Here, this mechanism is defined as the equivalent decrease mechanism. Meanwhile, the vast difference of the extinction intensities existing between LCP and RCP light as a function of *θ* indicates the existence of another mechanism, which causes the giant extrinsic chirality of the metal nanocrescent. In addition, we think the extrinsic chiral mechanism is that at a non-zero incident angle, the excitation efficiencies of LSPR modes for LCP and RCP light are different.

Next we describe the extrinsic chirality mechanism in detail. We give the rough optimum electric field distributions on the metal nanocrescent surface of circularly polarized light to excite the dipole mode (Fig. 3b) and tripolar mode (Fig. 3c). Under such electric field distributions, the excitation efficiencies of the dipole mode and tripolar mode are the highest for circularly polarized light. When $\,\theta =0^\circ$, the excitation efficiency is identical for circularly polarized light with opposite handedness. With the increase of *θ*, the electric field distribution on the metal nanocrescent surface of one circularly polarized light tends to approach the optimum electric field distribution, which results in an increase of the excitation efficiency. When the electric field distribution on the metal nanocrescent surface of this circularly polarized light matches well with the optimum one, the excitation efficiency is the highest. When *θ* continues to increase, the electric field distribution on the metal nanocrescent surface of this circularly polarized light starts to stay away from the optimum one, which results in the decrease of the excitation efficiency. For circularly polarized light with handedness opposite to mentioned above, however, case is different. At first, the electric field distribution on the metal naocrescent surface of this circularly polarized light tends to stay away from the optimum electric field distribution, which causes a decrease of the excitation efficiency. When *θ* continues to increase until it’s greater than a certain angle, the electric field distribution of this circularly polarized light starts to approach the optimum one, which results in the increase of the excitation efficiency. Here, the value of the critical angle mentioned above can not to be determined precisely in our work. The collective effect of the extrinsic chirality mechanism and the equivalent decrease mechanism leads to the results shown in Fig. 2c. The vast difference of the excitation efficiencies between the LCP and RCP light is formed and leads to the giant extrinsic chirality of the metal nanocrescent.

**Figure 4:**
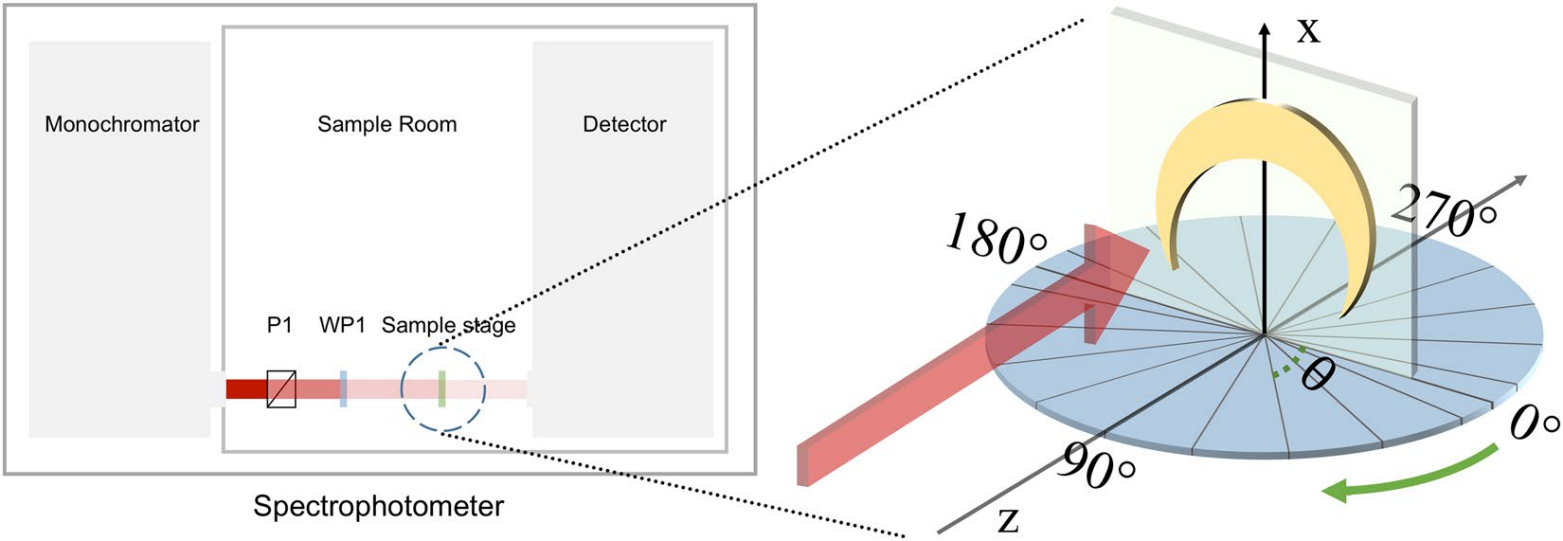
Schematic design of the experiment. Here, P1 stands for glan-taylor prism as a polarizer and WP stands for a wideband quarter-wave plat. The inset shows that the sample is mounted on a rotational micropositioning stage.

**Figure 5:**
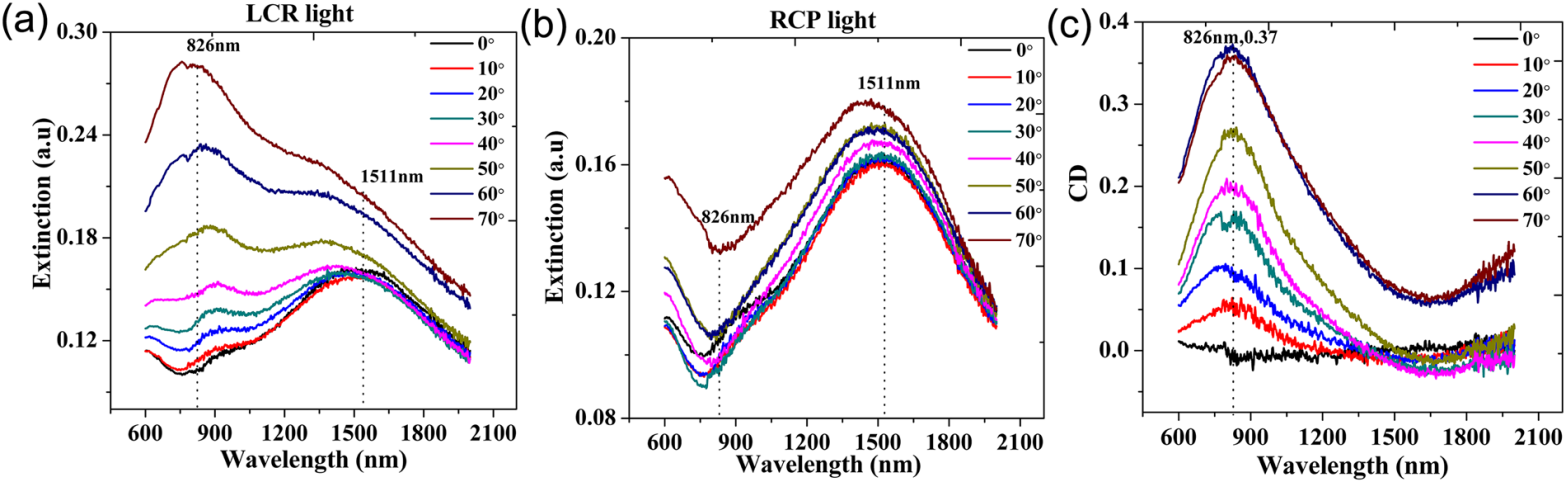
Experimental results of the nanocrescents (**a**) The extinction spectra of metal nanocrescents for LCP light with the increase of *θ*. (**b**) The extinction spectra of metal nanocrescents for RCP light with the increase of *θ*. (**c**) The calculated CD of metal nanocrescents with the increase of *θ* by experiment.

With numerical simulation providing fundamental feature of extrinsic chirality of the metal nanocrescent, we measure the spectra of large-area and monodisperse metal nanocrescents experimentally. To investigate the optical response, we measure its extinction using the experimental design, which is schematically plotted in Fig. 4. Details of the experimental design refer to METHODS. Figure 5a,b show the measured extinction spectra of the metal nanocrescents. Clearly, both spectra show two extinction peaks at around 1511 nm and 826 nm respectively. We consider that the peak at around 1511 nm corresponds to the dipolar mode and the peak at around 826 nm is the superposition of the tripolar and quadrupolar peaks. In our experiment the tripolar and quadrupolar peaks are not clearly distinguished. What’s more, we notice that there is a marked difference between experimental extinction spectra and the simulations ones, which is mainly due to the difference between the experimental setup and the simulation setup. Different from the simulation one, the number of the metal nanocrescents illuminated by the light increases with the increase of *θ* in experiments, and the increase is particularly prominent when *θ* is very large. This causes obvious differences between experimental and simulations results including the extinction and CD spectra. For example, the peak intensity (826 nm) excited by LCP light (Fig. 5a) keeps increasing with the increase of *θ* in experiment, which is different from the simulation results, that the extinction intensities firstly increase then decrease with the increase of *θ* for both the tripolar and quadrupolar peaks. Therefore, the difference of the experimental and simulation setup results in the difference of extinction spectra and CD spectra of experiments and simulations. The nanocrescents fabrication and experimental system need to be optimized in future research. In addition, the maximum CD measured in experiment (about 0.37) is achieved at 826 nm when $\theta =60^\circ$, which is different from the simulated one, $\theta =40^\circ$. As shown in Fig. 5b, with the increase of incident angle, the extinction peak located at 826 nm excited by RCP light weakens rapidly and then disappears in the large background signal. So, the maximum CD achieved in experiment at a larger incident angle than the simulated one. In spite of this, the experimental results are approximately consistent with the simulation ones, including the wavelengths of extinction peaks and CD peaks and the line-shapes of the extinction and CD spectra.

**Figure 6:**
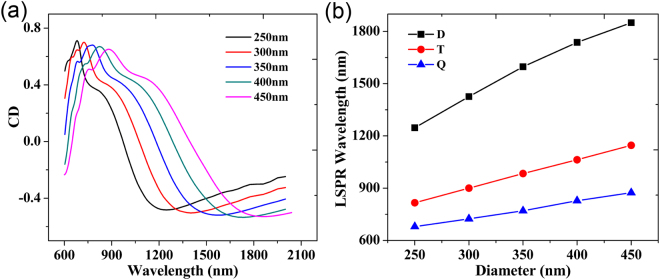
(**a**) Calculated CD spectra of the metal nanocrescents with the diameters over a range of 250–450 nm. (**b**) Dependence of LSPR wavelength of CD spectra on the metal nanocrescent diameter. Here the “D”, “T” and “Q” correspond to dipolar mode, tripolar mode and quadrupolar mode, respectively.

In addition, the optical response of the metal nanocrescent can be tuned via changing its diameter, which provides great benefit to application. The diameter of the metal nanocrescent is determined by the diameter of polystyrene (PS) spheres, which is commercially available with diameters over a range of 50 nm−10 *μ*m. We investigate the influence of metal nanocrescent diameter on the CD response by simulations. Figure 6a shows the CD spectra of the metal nanocrescents with diameters ranging from 450 nm to 250 nm when $\theta =40^\circ$. As the diameter becomes smaller, the resonance wavelengths of CD blue shift. A detailed description is shown in Fig. 6b. With the decrease of dimeter from 450 nm to 250 nm, the LSPR wavelength of dipole mode (black curve, Fig. 6b) blue shifts from 1850 nm to 1247 nm, the triploar one (red curve, Fig. 6b) blue shifts from 1146 nm to 816 nm, and the quadrupolar one (blue curve, Fig. 6b) blue shifts from 874 to 680 nm. Therefore, the CD spectra are sensitive to the nanocrescent diameter. And the resonance wavelength of CD can be tuned flexibly by changing the nanocrescent diameter.

## Conclusion

In conclusion, we report the strong extrinsic chirality of the large-area and monodisperse metal nanocrescents with giant and tunable CD effect. In the experiments and simulations results, the extinction spectra of the metal nanocrescent exhibit distinctly different for LCP and RCP light at a non-zero incident angle. And the resonance wavelengths of the giant CD locate at near the frequency of LSPR bands. Based on these results, we give a qualitative explanation for the giant extrinsic chirality of the metal nanocrescent, that the excitation efficiencies of LSPR modes for LCP and RCP light at a non-zero incident angle are different. The experimental results are basically consistent with the ones in simulations. The maximum CD, about 0.37, is achieved in the experiment. And the CD spectra can be easily tuned by changing the nanocrescent dimension. Benefitting from the simple and low-cost fabrication process, our work may help in promoting a better extrinsic chirality application.

## Methods

### Fabrication

The metal nanocrescents were fabricated using NL technology. The scheme of the fabrication process is shown in the Fig. 1a. At first glance, the 300 nm diameter PS spheres were randomly dispersed to a clear glass substrate pre-treated by oxygen plasma etching to form a submonolayer of spatially separated colloids. After that, the substrate was deposited by ion beam sputtering coating with a tilt angle of $\phi =45^\circ \,\,$to form a 50 nm thick gold layer. Next, the gold film layer was etched vertically by ion beam. At last, the PS spheres were cleaned by means of acetone solution.

### Experiments

The characterization of metal nanocrescents’ optical properties is implemented by a spectrophotometer (HITACHI U-4100). Figure 4 shows the diagram of the measurement. Circularly polarized light is achieved from monochromator in the spectrophotometer, through a polarizer (P1) and a wideband quarter-wave plat (WP). As the circularly polarized light irradiates the sample, the detector of the spectrophotometer receives the transmitted optical signal. Here, the symmetry axis of nanocrescent is parallel with the axis of rotation. The incident circularly polarized light (red arrow) is always perpendicular to the rotationally symmetrical axis of the metal nanocrescent. The angle *θ* is called the incident angle, which is tuned by the rotational stage. The stage is rotated clockwise and green arrows implies the direction. When ${\rm{\theta }}=0^\circ$, the surface normal of the metal nanocrescent is parallel to the propagation direction of the incident light.
